# Viewing Another Act as You Would Creates Altruistic Desires Towards that Other

**DOI:** 10.3389/fnhum.2017.00594

**Published:** 2017-12-08

**Authors:** Paul Bogdan

**Affiliations:** School of Information, University of Michigan, Ann Arbor, MI, United States

**Keywords:** social behavior, empathy, altruism, fairness, cingulate cortex, medial prefrontal cortex, temporoparietal junction, action selection

## Abstract

There has been growing evidence for the existence of distributed, frequently updating social “indices”, which are related to the reputation of others and predict altruism towards them. However, the means by which the brain modifies an index based on experiences is still unknown. This work utilizes recent insights on the role of the anterior cingulate cortex during perspective taking, dorsolateral prefrontal representations of context, the temporoparietal junctions relationship with understanding another’s background, and dorsomedial prefrontal activation patterns tracking reputation. It aims to show that cognitive empathy causes comparisons between a target’s action and the action one would wish to do in the target’s position. It also suggests that viewing a target perform the same action that one would in the target’s position creates altruistic desires towards the target. By considering these comparisons as central to understanding prosocial and antisocial motivations, a variety of behavioral studies are better explained. This piece seeks to open questions and discussions on the interplay of those brain regions, suggest future approaches to relationship therapy, and establish fundamentals for multi-agent models aimed at normative sociality.

## Introduction

Altruism is widely interpreted as taking actions for the benefits of another which confer a cost to oneself. While Charles Darwin initially found such behavior paradoxical, Nowak (2006) mathematically outlined situations where altruism is an evolutionarily beneficial aspect of cooperation. However, there still exists debate on how one’s cognitive processes give rise to such prosocial behavior. A systematic review identified explanations on the origins of altruistic behavior related to empathy, socially learned norms and fairness/reciprocation (Feigin et al., 2014). The concept of fairness/reciprocity are also closely associated with “reputation” (also referred to as status). In this context, the reputation of an individual or group determines the extent to which one values their benefits (Izuma, 2012). A recent review identified both competent and moral behavior as being common means of improving reputation (Bai, 2017). Notably though, members of a community may view the reputation of the same person differently. There does not yet exist a model for sociality which unifies or explains the above routes to altruism.

I argue that comparing how oneself and another would act for a given task is a fundamental aspect of social cognition, an outcome of cognitive empathy (theory of mind), and should be an element of unifying models. If the target would act how the judge (performing cognitive empathy) would wish to act in the target’s position, then the judge feels greater motivations to be altruistic towards the target. If target does not act how the judge believes would wish to act, particularly if the target is acting less altruistically than expected, then the judge will feel less altruistic desires towards the target. For instance, a judge may become upset that they were [cut off] by a target, while the target was <entering the highway>, the task. The judge’s anger towards the target is centered on the belief, that the judge would not [cut off] the target, if the judge was <entering the highway>. However, the target may believe they were justified as their task is <driving to hospital while wife is currently going through labor>. The upset judge would act the same if they understood the circumstances, but by neglecting the healthcare details, the judge believes the target has treated them *unfairly*. Moreover, if they knew the other was a student driver, this element, related to their background, would have made the other’s actions more acceptable. These examples show how antisocial desires emerge from seeing a target act not how the judge would. However, by changing the considered contextual behavior, the judge may realize that the target is acting how the judge would, creating prosocial desires.

To support the existence of such computations, I will first show how a view, which considers judges comparing the actions of targets to the fictive actions the judge would perform, as opposed to reciprocation theories (Levine, 1998; Behrens et al., 2009), affords explanations to paradoxical social psychology findings. Notably this includes results on defectors punishing cooperators in economic games, motor synchrony promoting altruism, and a preference for those who treat judges with fairness rather than favoritism.

To support this view’s neural plausibility, I will then outline findings on a cognitive empathy network involving the dorsolateral prefrontal cortex (dLPFC), temporoparietal junction (TPJ), anterior cingulate cortex gyrus (ACCg) and dorsomedial prefrontal cortex (dmPFC). The ACCg, which is known to assess the motivations of others (Apps et al., 2016), will be described as performing comparisons between one and another’s action plans. I will also show that the context of an action (<entering highway> vs. <entering highway while wife is in labor>) is represented by the dLPFC, that accounting for the differences between the backgrounds of others (driver vs. student driver) utilizes the TPJ, and that indices which represent the extent to which a specific other’s benefits are valued are represented by the dmPFC.

## Reputation Indices Define The Extent to Which Another’s Benefits Are Valued

Nowak (2006) summarized findings showing that altruistic tendencies can evolve if they are directed at others who one expects will eventually reciprocate the altruism. Moreover, the extent of reciprocation expected determines the strength of one’s altruistic tendencies, which are defined by the amount of benefits the other receives relative to the cost one is willing to incur. However, direct reciprocity is dependent on repeated encounters with the same individual and does not explain the advantages of altruistic tendencies towards those who are generally helpful in one’s community, indirect reciprocity. Nowak (2006) elaborated that the concept of reputation accounts such intercommunity altruism between donors and recipients who may never directly interact again.

The following formalism is also provided, showing that an individual should perform an altruistic act when another’s reputation is greater than the ratio of the costs one incurs relative to the benefits another incurs, *r* > *c*/*b*. This view can also be interpreted as one receiving utility equal to *r* * *b* when helping another (Levine, 1998).

Notably, people may view the reputation of the same community member differently causing varying altruistic behavior towards that member. Levine (1998) also showed that it is common for people to perceive the reputation of novel others as being greater than zero, *r* > 0, creating light altruistic tendencies towards people one is unfamiliar with. Reputation indices may also be less than zero and promote spiteful actions which cause a large cost to another at a smaller cost to oneself. Spiteful actions are beneficial for one’s own interests as they coax defectors into cooperating for the benefits of others (Herrmann et al., 2008; Starmans et al., 2017). Both prosocial and antisocial reputation indices have been found to exist in the brain and are frequently updating and distributed entities (Parkinson et al., 2017). This article proceeds concerned with how reputation indices change.

## Cognitive Empathy Assesses Deservingness

A meta-analysis revealed that higher cognitive empathy scores were correlated with prosocial behavior (Imuta et al., 2016). Although, the correlation with altruism was weak (*r* = 0.19, *p* < 0.05, 37 studies sampled) and a number of the studies found a lack of effect (Imuta et al., 2016). Correlations between cognitive empathy and antisocial behavior have also been found (Cowell et al., 2015).

I interpret these findings as suggesting, that* cognitive* empathy does not necessarily promote altruism, but performs computations to assess the reputation/deservingness of others. This conclusion is supported by cognitive empathy questionnaire scores predicting the extent to which one is sensitive to justice (Decety and Yoder, 2016). Justice sensitivity predicts the extent to which one will reciprocate the altruism of others by modulating the rate of reputation index change following another’s prosocial or antisocial actions (Levine, 1998). The expected behavioral outcomes of this relationship between cognitive empathy and changing reputation indices indeed occur. Greater cognitive empathy was found to promote altruistic offers in ultimatum games only when their partner made fair offers previously (Schug et al., 2016). Greater cognitive empathy also affects rejection rates of ultimatum games (spiteful actions; Beadle et al., 2012). Notably, young adults showing greater cognitive empathy will decrease their rejection rate (an altruistic shift) after viewing their partner be selfish, while older adults will increase their rejection rate (a spiteful shift; Beadle et al., 2012). Such results are understandable given our view of altruistic desires emerging from judging another to be acting similarly to how one would in their position, as a different study found that young adults are more selfish during ultimatum games than older adults (Bailey et al., 2012).

This piece proceeds showing how further social psychology findings are well explained using a view which predicts altruistic desires towards those acting how one would act. Explanations on altruism due to affective empathy (Feigin et al., 2014) utilize different neural processes (Kanske et al., 2016) and are not considered. Similarly, explanations pertaining to social norms (Feigin et al., 2014) also involve different neural circuits than those related to reputations (where the utility of another is treated as one’s own; Ruff et al., 2013; Parkinson et al., 2017). The remaining explanations, as identified by recent reviews on altruism and reputation (Feigin et al., 2014; Bai, 2017), are concerned with how performing fair/moral actions increases one’s reputation. Such views relate closely to positions stating that one feels altruistic desires towards those that will reciprocate (Levine, 1998; Nowak, 2006). The following section explains how a variety of findings on fairness are equally or better explained using this work’s focus on action comparisons and what the judge would do in the other’s position.

## The Meaning of Fairness

Perceiving a partner in an economic game as being fair creates altruistic desires towards that person (Rabin, 1993; Levine, 1998; Herrmann et al., 2008). Similarly, perceiving a partner to be unfair prompts spiteful actions towards them (Nowak et al., 2000; Forber and Smead, 2014). Moreover, viewing a target to be fair or unfair, even when one is an unaffected third party, also affects valuations of the other’s utility (Fehr and Fischbacher, 2004). This relationship has been identified in third-party subjects as young as 15-months old (Schmidt and Sommerville, 2011). Fairness promoting altruism also manages to explain altruistic behavior better than purely reciprocation-based theories, as 6–8-year old subjects, when comparing a distributor who shared toys equally among the subject and others with another distributor who showed favoritism towards the subject, were split 50/50 in terms of which distributor they preferred (Shaw et al., 2012).

However, it prompts the question, “what is fairness?”. In ultimatum/dictator games, fairness is straightforward: both players receive similar amounts of money (Tam, 2014). Although outside the laboratory, a consensus will not be so easily found, and even in ultimatum games, receiving subjects deem situations where they are given more money to be fairer than those where their partner receives additional money (Tam, 2014). This suggests fairness is subjective, often self-serving and without consistency (Konow, 2005; Feng et al., 2013).

To account for this, I believe biologically concerned positions on assessing the fairness of others, should deem that another’s action is “fair” (promoting of altruistic desires) if it is what one *perceives* one would do in the other’s position. The element of perception (and not having to actually perform) also allows prescriptive biases to enter.

A variety of findings on fairness can be explained utilizing this other-perspective, action comparison view. For instance, studies have shown that participants will utilize self-sacrificing punishments to coax others into cooperating during economic games (Boyd and Richerson, 1992; Herrmann et al., 2008). In these studies, spiteful actions are usually administered by cooperators towards defectors due to unfair defector behavior (the defectors are not acting how the cooperators would). However, Herrmann et al. (2008) paradoxically found that in some cultures, defectors will punish cooperating partners. Such results are inexplicable by reciprocation theories. However, a view which predicts antagonistic intentions towards those who act differently than one would in their position can better explain Herrmann’s et al. (2008) findings.

Levine’s (1998) model, which proposed that one acts altruistically towards another whom one perceives as altruistic, is an example of a reciprocation model struggling to account for all findings. Such findings also include Nowak’s et al. (2000) study, which found that those who accepted unfair offers garnered lower reputation and received more abuse. This conflicts with Levine’s (1998) stance that their altruistic, forgiving behavior should beget a higher reputation (reviewed by Bai, 2017). In Nowak’s et al. (2000) work, those who received abuse were *not* acting as the judging abuser would have acted in their position (and retaliate via punishment). By understanding the victim’s actions in this expectations view, the continuing abuse becomes clearer.

## Other Social Psychology Findings in This Context

The centrality of sharing views may explain why apologies are particularly powerful when the target assures the victim, that the action will not occur again (Scher and Darley, 1997). Such apologies, if believed, result in the victim perceiving themselves and the target to have similar views on the acceptable actions for a situation.

Viewing another as having synchronous motor activity with one is another means of perceiving them to be performing similar actions. This type of action comparison intuitively is unrelated to altruistic motivations, but motor synchrony (walking in-step vs. not or mouthing the words to a song in sync vs. out of sync) was indeed found to promote altruism (Wiltermuth and Heath, 2009). In support of these findings being due to cognitive processes, Wiltermuth and Heath (2009) also showed altruistic motivations not to be associated with emotional responses. Similar relationships between altruism and synchrony were confirmed in 14-month infants (Trainor and Cirelli, 2015).

Further evidence for the behavioral claims emerges from its proposed routes to conflicts (not having similar views on a what action is best for a task) being common issues relationship therapy methods address. For instance, most couples therapy models include focuses on five points: each member understanding the relationship’s role in the other’s life, identifying the effects of actions on the other, not avoiding emotions regarding the relationship, effective communication, and vocalizations of appreciations (Benson et al., 2012). The successes of these approaches can all be represented in the proposed similarity view: discussing the role of the relationship aligns context representations/norms (which allow rational conclusions of appropriate actions), identifying effects on others aligns valuations (allowing similar decisions on what actions are appropriate), and avoiding emotions prevents effective communication. Communication is necessary to align tasks and action valuations. Action valuations are also better aligned by emphasizing positives.

These approaches aim to address failures to fully take the other’s perspective. Coplan (2011) elaborates on the meaning of other-oriented perspective taking, as opposed to self-oriented perspective taking. The latter only involves one putting themselves in the other’s context without accounting for differences in life histories/backgrounds, which may result in different competencies, assets or valuation schemes. Other-oriented perspective, on the other hand, accounts for these but requires greater knowledge of the other and cognitive effort.

The differences Coplan (2011) describe are useful for tying the neural relationships between the TPJ and dLPFC to behavioral outcomes. The following sections elaborate on these and other neural findings from the regions involved in cognitive empathy, the dmPFC, ACCg, TPJ and dLPFC, and how these findings support a view on perspective taking comparisons promoting altruism.

## Altruistic Tendencies Are Encoded by dmPFC Indices

The dmPFC is understood as highly relevant to social cognition. Greater dmPFC activation occurs when subjects are told to think about others, in contrast to vmPFC, dLPFC and left insula activations, which all were greater during self-thinking (Denny et al., 2012). Increased dmPFC activation also wasn’t exclusive to direct viewing of others, but also occurs when viewing video clips (Iacoboni et al., 2004) or images (Wagner et al., 2011) of social interactions.

Its activation is expected to be for the assessment of reputations, as dmPFC activation tracks cues for assessing reputations (Muscatell et al., 2012) and changes in self-esteem due to social evaluation (Eisenberger et al., 2011). Related to this role, the dmPFC also shows greater activation when witnessing unfair offers (Cheng et al., 2017), when learning norms or when witnessing the violation of norms (Berthoz et al., 2002), when realizing that a social group does not share your opinion (Klucharev et al., 2009), or when realizing the advice of another is bad (Behrens et al., 2008). Also, supporting the relationship between deservingness and reputation, medial prefrontal cortex (mPFC) activations were found to encode judgments of another’s deservingness, features notably not encoded by the TPJ (Tusche et al., 2016).

As expected given the relationship between reputation and altruism, dmPFC activation was also found to be correlated with both altruistic intentions (Rilling et al., 2008; Waytz et al., 2012) and punishment of others at a cost to oneself (spite; Feng et al., 2016). This has given rise to explanations that the dmPFC’s role in altruism is encoding reputation indices, *r*, and modulating activity in the vmPFC (Izuma, 2012), which encodes the rewards associated with an action, *r* * *b* (Weilbächer and Gluth, 2016; Hunt and Hayden, 2017). More recent developments have refined the relationship between the dmPFC and vmPFC, showing that reputation indices of higher valued others are located more ventrally in the mPFC, where purely selfish rewards exist (Sul et al., 2015).

ACCg activation similarly reflects beliefs of another’s reputation (King-Casas et al., 2005), and its activation reflected learning of social hierarchies (Qu et al., 2017). These findings, identification of reciprocal projections between the ACCg and dmPFC (Apps et al., 2016), along with projections from the dLPFC and TPJ to the ACCg communicating information necessary for perspective taking (Lockwood et al., 2015) have given rise to explanations that the ACCg is performing computations allowing encoding of reputations in the dmPFC (Behrens et al., 2009; Apps et al., 2016). These findings and their relations to a general ACC role in decision-making are elaborated in the next section.

## Action Comparisons in The ACC

The dorsal ACC (dACC; also found referred to as the ACC sulcus) is expected to have a role in comparing possible actions for a task (Hayden et al., 2009; Kolling et al., 2016), as the dACC’s activation patterns reflect the valuations of actions (Alexander and Brown, 2011) and non-reward state transformations (Hayden and Platt, 2010) of not only performed but also of fictive decisions (Hayden et al., 2009; Kolling et al., 2016). For these functions, the dACC densely shares reciprocal connections with mPFC and lateral PFC (LPFC) regions (Beckmann et al., 2009; Medalla and Barbas, 2010). Such findings make ACC activation being correlated with regret, cognitive dissonance, and attitude change unsurprising (van Veen et al., 2009). Rangel and Hare (2010) and Cisek (2012) offer richer reviews of ACC comparisons and the existence of valuations in an action-space.

The ACCg has similar properties as the dACC, but for social assessments of another’s actions, which I argue occur during cognitive empathy. Such a parallel is understandable in the context of the ACC as an action assessment module if empathy is tempered with regards to perception-action coupling (Preston and de Waal, 2002) and the Empathy Simulation Hypothesis (Gallese and Goldman, 1998). Perception-action coupling involves the same neurons representing an action to be also activated when viewing another perform said action (Preston and de Waal, 2002). Such findings have given rise to simulatory explanations for empathy where empathy is explained as simulating oneself in another’s situation (Gallese and Goldman, 1998).

Beyond previously mentioned findings, there exist further results showing that the ACCg is a social parallel to the dACC, where the actions of others are compared to the fictive actions the judge believes are ideal given the other’s position and background. ACCg activations have been found to reflect the rewards, pains, and net value another receives for both the actions one performs (Apps and Ramnani, 2014) and actions the other performs (Apps et al., 2016). Notably, such reward judgments utilize expected valuations from the other agent’s reference frame (Apps et al., 2016), which requires information from the TPJ and dLPFC to understand.

## Context and Reference Frame Encodings by The dLPFC and TPJ

The dLPFC shows increased activation when assessing the appropriateness of another’s action (Buckholtz et al., 2008), as it represents the other’s context (and the morals/norms/expectations associated with such a context) during action assessment. Support for this extensive. Disruption of the dLPFC reduces norm sensitivity (Buckholtz et al., 2015) and impairs the ability to account for the others’ context during moral judgment (Tassy et al., 2011). Disruption of the right dLPFC results in lower rejection rates of unfair ultimatum game offers (Knoch et al., 2006). Increased dLPFC activity is also associated with better accounting of context in a variety of non-social paradigms (Ruff et al., 2013; Smittenaar et al., 2013; Rudorf and Hare, 2014; Balderston et al., 2017).

Although among social paradigms, many which identify relationships between dLPFC activation and behavioral outcomes found similar correlations with TPJ activation. For instance, dLPFC lesioned patients and low TPJ gray mass volume subjects both do worse in the eyes test for assessing theory of mind capabilities (Geraci et al., 2010; Sato et al., 2016). dLPFC and TPJ increased activations are also both correlated with better understandings of intentionality (Güroğlu et al., 2011), and flexibility during moral prescription tasks (Tei et al., 2017).

However, there exist experiments where the two regions perform differently, showing that the TPJ plays a role in representing another’s unique background. While dLPFC activity will track the behavior of computers (Seo and Lee, 2008), TPJ activity is largely exclusive to interpreting humans and does not change from baseline due to computer responses (Lee and Harris, 2013). Further evidence for this role in mentalizing others is extensive: greater TPJ activation predicts the degree of cognitive other-perspective taking (Tusche et al., 2016), transcranial DC stimulation of the TPJ also increases one’s capabilities to imitate another (Hogeveen et al., 2015) and electrical stimulation of the right TPJ was found to induce out-of-body experiences (Blanke et al., 2002).

Most relevant for action judgment, during assessment of blameworthiness: TPJ activity peaks prior to dLPFC activation, suggesting distinct roles for the two regions or a modulatory/filter role for the TPJ (Buckholtz et al., 2008), and increased dLPFC activity was associated with another’s circumstances being deemed the cause of another’s actions, while TPJ activation correlated with the other’s character being the cause (Berthoz et al., 2002). Coplan (2011) provides a review of the behavioral manifestations of accounting for someone’s context instead of their context and background.

Together, the above findings on the dLPFC and TPJ show the existence of context and background representations, which provide necessary information for ACCg comparison computations that change the values of reputation indices, *r*, in the dmPFC.

## Concluding Remarks

I aimed to show that a necessary aspect of cognitive empathy is comparing the actions/thoughts of another to one’s own actions/thoughts. By reinterpreting behavioral findings, I highlighted how altruistic desires can be reasonably hypothesized as occurring due to viewing another as acting as one would. A formal model is not proposed here, but I hope future works addresses the importance of comparisons between another’s action and one’s own action plans in models of sociality.

## Author Contributions

PB is the sole author and is responsible for the reading, writing and editing.

## Conflict of Interest Statement

The author declares that the research was conducted in the absence of any commercial or financial relationships that could be construed as a potential conflict of interest.
